# The role of Probiotics in allergic diseases

**DOI:** 10.1186/1710-1492-5-5

**Published:** 2009-10-22

**Authors:** Sonia Michail

**Affiliations:** 1 Department of Gastroenterology and Nutrition, Wright State University Boonshoft School of Medicine Dayton Children's Medical Center, Dayton, Ohio 45404, USA

## Abstract

Allergic disorders are very common in the pediatric age group. While the exact etiology is unclear, evidence is mounting to incriminate environmental factors and an aberrant gut microbiota with a shift of the Th1/Th2 balance towards a Th2 response. Probiotics have been shown to modulate the immune system back to a Th1 response. Several in vitro studies suggest a role for probiotics in treating allergic disorders. Human trials demonstrate a limited benefit for the use of probiotics in atopic dermatitis in a preventive as well as a therapeutic capacity. Data supporting their use in allergic rhinitis are less robust. Currently, there is no role for probiotic therapy in the treatment of bronchial asthma. Future studies will be critical in determining the exact role of probiotics in allergic disorders.

## Introduction

Currently, an estimated 20% of the population worldwide is suffering from some form of allergic disorder with a prevalence that continues to rise [1]. For example, the prevalence of childhood asthma in the USA increased by 50% from 1980 to 2000 [2]. Atopic diseases involve Th2 responses to allergens [3]. These clinical disorders are characterized by immediate hypersensitivity.

Although the exact etiology of allergic diseases remains ambiguous, many investigators have proposed that environmental exposures may be major trigger factors in the development of allergic diseases. As the rise in prevalence of allergic diseases has been seen mostly in industrialized countries, this led investigators to formulate the hygiene hypothesis in an attempt to explain the basis of the disease. This hypothesis entails that reduced family size and childhood infections have lowered our exposure to microbes, which play a crucial role in the maturation of the host immune system during the first years of life[4].

In addition to environmental factors, the intestinal flora may be a contributor to allergic disease due to its substantial effect on mucosal immunity. Allergic responses are thought to arise if there is absence of microbial exposure while the immune system is still developing [5,6]. Exposure to microbial flora early in life allows for a change in the Th1/Th2 balance, favoring a Th1 cell response. Several reports suggest that the make-up of intestinal microflora can be different in individuals with allergic disorders and in those who reside in industrialized countries where the prevalence of allergy is higher [7-9]. For example, children from an industrialized country like Sweden harbor less *Lactobacilli *and *Bifidobacteria *(and more *Staphylococcus aureus *and *Clostridia*) in their bowels in comparison to children who live in countries like Estonia where allergic disorders are not as common [10-12].

The concept that children with allergic disorder harbor a different profile of microflora has been supported by several other studies [8,13-17]. Perhaps the most convincing of these is the KOALA study, which examined flora of 957 infants in the Netherlands [18]. The study revealed that *C. dificile *colonization at one month of age was associated with an increased likelihood of eczema, recurrent wheezing, and atopic dermatitis. *E. coli *colonization was associated with eczema rather than recurrent wheezing or atopic dermatitis. No association with *bifidobacteria *colonization, *B. fragilis *or *lactobacilli *colonization was observed.

While this concept has been validated in several other studies, there are a few reports that do not show a significant difference in microflora composition. A recent study comparing microflora composition of 324 European infants showed no association between food sensitization or atopic dermatitis and the intestinal bacteria [19]. In general, however, most studies suggest that an association exists.

### Mechanisms of action of probiotics in allergic disorders

The United Nations Food and Agricultural Organization and the World Health Organization define probiotics as "live microorganisms, which, when administered in adequate amounts, confer a health benefit to the host" [20]. Prebiotics are defined as non-digestible oligosaccharides, such as fructo-oligosaccharides and trans-beta-galacto-oligosaccharides, that selectively stimulate the growth of bifidobacteria and lactobacilli, thus producing a prebiotic effect. Synbiotics is a term referring to the use of both prebiotics and probiotics simultaneously.

As described above, allergic disorders are associated with a shift of the Th1/Th2 cytokine balance towards a Th2 response. This leads to activation of Th2 cytokines and the release of interleukin-4 (IL-4), IL-5, and IL-13 as well as IgE production [21].

Probiotics can potentially modulate the toll-like receptors and the proteoglycan recognition proteins of enterocytes, leading to activation of dendritic cells and a Th1 response. The resulting stimulation of Th1 cytokines can suppress Th2 responses [21]. Pediatric studies suggest that probiotic use in children with atopic conditions such as atopic dermatitis results in enhancement of IFN-production and decrease d IgE and antigen-induced TN F-, IL-5, and IL-10 secretion [22-24].

### The role of probiotics in allergic disorders

The interest in probiotic therapeutic potential in allergic disorders stemmed from the fact that they have been shown to reduce inflammatory cytokines and improve intestinal permeability in vitro. Such effects would be desirable in treating allergic disorders. Therefore, several studies have been designed to examine the efficacy of probiotics in many allergic conditions, such as eczema, allergic rhinitis, asthma and food allergies.

#### a) Role of probiotics in atopic dermatitis

Several human trials, as well as, numerous animal and in vitro studies suggest a beneficial effect of probiotics in allergic diseases. The therapeutic and preventive role of probiotics in atopic dermatitis has been extensively studied.

#### 1. Prevention of atopic dermatitis

The prevention of allergic diseases relies heavily on preventing sensitization to an offending allergen. Enomoto and colleagues investigated the association of consumption of fermented dairy products and the development of allergy and allergic sensitization in Japanese students as reflected on serum levels of total IgE values, specific IgE to house dust mite and Japanese cedar pollen. The report demonstrated a significant reduction in allergy development among the students consuming fermented milk in comparison with students who did not consume fermented products[25].

The effect of probiotics on preventing atopic dermatitis has been demonstrated in randomized studies from Finland where *Lactobacillus *GG or placebo was given to pregnant mothers with a strong family history of eczema, allergic rhinitis or asthma, and to their infants for the first six months after delivery. The frequency of developing atopic dermatitis in the offspring was significantly reduced by 2, 4, and 7 years [26-28], by 50%, 44%, and 36% respectively.

Similar studies have yielded comparable results. The use of the probiotic *E-coli *in the early postnatal period decreased the incidence of serum specific IgE allergies at 10 and 20 years of age in a long-term prospective study [29,30]. Other studies could only relate probiotic benefits to a certain subset of dermatitis patients. The incidence of IgE-associated dermatitis, rather than other types of atopic dermatitis, was decreased after the oral consumption of probiotics, namely *L. reuteri *or a mixture of four probiotic bacteria and prebiotics [31,32]. However, Taylor et al could not confirm such effects in a randomized placebo-controlled double-blind study. *L. acidophilus *did not decrease the risk of developing allergy in a large number of infants[33,34]. There were two major differences between Taylor^D^s study and the others. The type of probiotic product was different as well as the timing of the introduction of the probiotic, Taylor et al administered the probiotic supplement postnatally, while other studies administered probiotics before and after birth. Prenatal supplementation may prove to be crucial for the preventive benefit of probiotics in this disorder.

Prebiotic oligosaccharides have also been shown to reduce the incidence of atopic dermatitis when given to infants at risk for atopy during the first six months of age[35]. No comparisons made to probiotics and have not been used prenatally.

A recent Cochrane review meta-analysis found a significant reduction in the likelihood of developing infant eczema with the use of probiotics. The meta-analysis described five studies enrolling 1477 infants. However, the authors found significant heterogeneity and with further focused on children with dermatitis that have positive skin prick test or specific IgE sensitization, there were no significant benefits noted with probiotics. The authors concluded that there was no current evidence to support the administration of probiotics to prevent eczema and recommended further studies to determine reproducibility[36].

In general, the role for probiotics in the prevention of atopic dermatitis (table 1) awaits future studies.

**Table 1:** Prevention of sensitization and allergic diseases

**Author**	**Year**	**Study type**	**Probiotic type**	**Results**
Abrahamsson	2007	R, C, DB	*L. reuteri*	Decreased IgE-associated eczema

Kalliomäki	2007	R, PC, DB	LGG	Decreased atopic dermatitis

Kukkonen	2007	R, PC, DB	LGG, *L. rhamnosus *LC705, *B. breve *Bb99, *P. freudenreichii *ssp shermanii JS	Lower IgE-associated diseases and eczema

Taylor	2007	R, PC, DB	*L. acidophilus *LAVRI- Al	No change in atopic dermatitis rates or SCORAD

Taylor	2007	R, PC, DB	*L. acidophilus *LAVRI- Al	No change in atopic dermatitis

Lodinova- Zadnikova	2004	C	*E. coii*	Decreased long-term incidence of allergy

Kalliomäki	2003	R, PC, DB	LGG	Decreased atopic dermatitis

Lodinova- Zadnikova	2003	C	*E. coii*	Decreased allergy development

Kalliomäki	2001	R, PC, DB	LGG	Decreased atopic dermatatis

Rautava	2001	R, PC, DB	LGG	Decreased atopic dermatitis

R = randomized, C = controlled, PC = placebo-controlled, DB = double-blinded, LGG = Lactobacillus GG, SCORAD = severity scoring of atopic dermatitis index

#### 2. Treatment of atopic dermatitis

Once allergic diseases develop, one goal of therapy is to control the patient's clinical symptoms. Probiotics may help to decrease the severity of atopic dermatitis and food allergy. Most clinical studies have targeted pediatric patients (table 2).

**Table 2:** Probiotics in treatment of allergies

**Author**	**Year**	**Study type**	**Probiotic type**	**Results**
Giovannini	2007	R, PC, DB	*L. casei *DN-114001	Longertime free from asthmaIrhinitis episodes Less number of episodes of rhinitis episodes

Tamura	2007	R, PC, DB	*L. casei *strain shirota	No change in allergic rhinitis

Xiao	2007	R, PC, DB	*B. Ion gum *BBS36	Ameliorate Japanese cedar pollinosis

Brouwer	2006	R, PC, DB	*L. rhamnosus *I LGG	Lower SCORAD (no different from placebo)

Fälster-Holst	2006	R, PC, DB	*L. rhamnosus *GG	Lower SCORAD (no different from placebo)

Passeron	2006	R, PC, DB	*L. rhamonosus *Lcr35 and prebiotic	Decreased SCORAD

Sistek	2006	R, PC, DB	*L. rhamnosus*, B. lactis	Decreased SCORAD

Xiao	2006	R, PC, DB	*B. Iongum *BBS36	Ameliorate Japanese cedar pollinosis

Xiao	2006	R, PC, DB	*B. Iongum *BBS36	Ameliorate Japanese cedar pollinosis

Ciprandi	2005	C	Bacillus clausii spores	Decreased nasal symptoms

Ishida	2005	R, PC, DB	*L. acidophil us *L-92	Decreased nasal and ocular symptoms

Peng	2005	R, PC, DB	*L. plantar um *33	Decreased perennial allergic rhinitis

Viljanen	2005	R, PC, DB	LGG or MIX	Decreased SCORAD

Weston	2005	R, PC, DB	*L. ferment um*	Decreased SCORAD

Rosenfeldt	2004	R, PC, DB	*L. rhamnos us *and *L. re uteri*	Decreased frequency of gastrointestinal symptoms and lactulose:mannitaol ratio

Wang	2004	R, PC, DB	*L. paracasei*	Decreased frequency and level of bother of allergic rhinitis

Hattori	2003	C	*B. breve *M-16V	Ameliorate cutaneous and allergic symptoms

Kirjavainen	2003	R, PC, DB	LGG	Decreased SCORAD

Rosenfeldt	2003	R, PC, DB	*L. rhamnos us *and *L. re uteri*	Decreased SCORAD

Helin	2002	R, PC, DB	LGG	No effect on birch pollen allergy

Isolauri	2000	R, PC, DB	*B. lactis *(Bb-12) or LGG	Decreased SCORAD

Majamaa	1997	R, PC, DB	LGG	Decreased SCORAD

Wheeler	1997	R, PC, DB	*L. acidophil us*	Increased IFN-and less eosinophilia

R = randomized, C = controlled, PC = placebo-controlled, DB = double-blinded, LGG = Lactobacillus GG, SCORAD = severity scoring of atopic dermatitis index

In 1997, the first published study in this area [37] examined the effect of *Lactobacillus GG *in mild atopic eczema in a modest number of infants. After four weeks, SCORAD scores dropped from 26 to 15, while the control group only changed from 21 to 19. However, one month after the probiotic was discontinued, both groups had comparable SCORADs. Therefore, in this particular study, the effect of the probiotic was short lived.

The same group of investigators subsequently published two additional studies. One study, published in 2000[38], compared *Lactobacillus GG*, or *Bifidobacterium lactis *Bb-1 2 to placebo. After two months SCORAD scores decreased from a baseline of 16 to 1, 0, and 13.4 respectively. However, after 6 months, the median SCORAD was zero (0-6.6) in all groups, suggesting that the probiotic effect is limited to rapid initiation of improvement in mild disease. The other study underscored the importance of viability [39]. While live probiotic administration resulted in statistically significant improvement of scores, the use of heat-inactivated *Lactobacillus GG *was associated with adverse gastrointestinal symptoms and further study enrollment was thus halted.

Another study by Kirjavainen et al suggested that *Bifidobacterium lactis *Bb12 modifies gut microflora to alleviate early onset atopic eczema[7]. A randomized, but not placebo-controlled study suggested that synbiotics and prebiotics significantly improve atopic dermatitis in older children [40].

The largest study (n = 230) to date, compared the effects of *Lactobacillus GG*, a probiotic mix, or placebo[41]. There was no difference between the groups after 4 weeks of therapy, or4 weeks after study supplement was discontinued. However, infants receiving *Lactobacillus GG *who had specific IgE sensitization had a greater reduction in SCORAD when compared to the placebo group (-26.1 versus -19.8, p = 0.036). Furthermore, a reduction of TNF-alpha and an increase in fecal IgA levels were noted [42]. Two other studies demonstrated comparable results. A study by Rosenfeldt et al. from Denmark used lyophilized *Lactobacillus rhamnosus *19070-2 and *Lactobacillus reuteri *DSM 122460) in older children (average age 5.2 years), and statistically significant improvement in SCORAD was only seen in a subset of children with positive skin prick test and elevated IgE levels [43]. Another study by Sistek et al. showed efficacy of the probiotic *Lactobacillus rhamnosus *and *Bifidobacteria lactis *in food-sensitized children[44]. Those three studies taken together reveal that probiotics were not effective for all children with atopic dermatitis but rather in the subset of IgE sensitized children.

However, a study from the Netherlands by Brouwer et al.[45] and another study from Germany by Folster-Holst et al.[46] showed no effect of *Lactobacillus rhamnosus *or *Lactobacillus GG *in infants with atopic dermatitis regardless of their IgE sensitization status.

In 2005, Weston et al.[47] from Australia published their experience with using *Lactobacillus fermentum *VRI-003 PCC for 8 weeks in 53 infants with atopic dermatitis. After 16 weeks the probiotic group had significant reduction of SCORAD scores (p = 0.03) while the placebo group did not (p = 0.83). However, while the change in SCORAD scores from baseline in the probiotic group was significant, the difference between the probiotic and placebo group did not quite reach statistical significance (p = 0.06) by the 16^th ^week.

As pointed out by Passeron and Lacour, in their letter to the editor[48], children receiving placebo treatment in many of these studies significantly improved within a much shorter than expected time. Cellulose and maltose dextran were used as placebo, which could have a prebiotic effect thus explaining the improvement seen in the placebo group. To further examine this effect, the same investigators compared the effects of prebiotics and probiotics (synbiotics) versus prebiotics alone and concluded that both groups had a significant reduction in the SCORAD scores after 3 months[40].

The most recent randomized trial was designed to investigate the therapeutic benefit of Lactobacillus rhamnosus GG (LGG) in infants with atopic dermatitis. Infants 3-12 months of age with mild-to-moderate atopic dermatitis were randomized to receive LGG or placebo as a food supplement for 12 weeks. Fifty-four infants received LGG and 48 infants received placebo. Symptoms improved overtime after 4, 8, and 12 weeks, without any group being statistically different [49].

A recent meta-analysis suggested that probiotics may benefit children and infants with the disorder [50]. The meta-analysis identified ten randomized, controlled trials. A significant overall benefit was demonstrated after the use of probiotics, resulting in a reduction of the dermatitis scores (SCORAD) compared to placebo. *Lactobacillus GG *appeared to be more effective than other probiotic preparations and children with more severe disease were more likely to benefit from the use of probiotics. Another recent meta-analysis did not show a therapeutic difference among children receiving probiotics [51]. However, this analysis excluded six of the ten studies published, making the validity of the report questionable.

Other studies have examined the effect of probiotic consumption on sensitization to several allergens (e.g. peanut, hen's egg, soy, wheat, milk, cat, dog), as determined by specific IgE production or skin prick test reaction (SPT). The authors could not find a difference before and after the treatment[22,26,45].

Taken together, some of these studies show a slight benefit over placebo for the treatment of atopic dermatitis. However, several of the studies show no benefit.

#### b) The role of probiotics in Asthma

A small number of studies exist that try to address the efficacy of probiotic supplementation in the treatment or prevention of asthma. Such studies have heavily focused on the treatment rather than prevention of asthma. Perhaps the largest and the most recent trial was conducted by Giovannini and colleagues using fermented milk containing *Lactobacillus casei *and studying its effect on the number of episodes of asthma and allergic rhinitis[52]. One hundred and eighty seven children, between two and five years of age, were included in the study. At the end of the twelve-month trial period the investigators found no statistical difference between intervention and control groups of asthmatic children. However, the number of rhinitis episodes was lower in the probiotic group leading the authors to conclude that *Lactobacillus casei *may benefit children with allergic rhinitis but not asthmatic children.

One randomized placebo-controlled crossover study examined the effect of yogurt containing *S. thermophilus *and *Lactobacillus bulgaricus *when given with or without *Lactobacillus acidophilus *to adolescents and adults with asthma who were sensitized to inhalant allergens. There was no difference in clinical parameters of asthma or laboratory markers of inflammation[53]. One concern with this experimental design arises from the fact that the placebo group received yogurt mixed with bacteria that have probiotic properties. At any rate, neither the active group nor placebo had any improvement in lung function.

The efficacy of probiotics in asthma as a preventive measure has not been evaluated and may be worthwhile studying. However, to date there is no evidence to justify the use of probiotics for treatment or prevention of asthma.

#### c) The role of probiotics in Allergic Rhinitis

Reports on the efficacy of probiotics in treating allergic rhinitis are conflicting. Some studies suggest efficacy such as the study by Wang and colleagues, where *Lactobacillus paracasei-*33 was given for 30 days to 80 children with perennial rhinoconjunctivitis. The quality of life questionnaire scores significantly improved relative to placebo[54]. A Japanese study demonstrated that intake of *Bifidobacterium lon gum *BB536 as a yogurt supplement is effective in relieving symptoms of Japanese cedar pollinosis[55,56]. Furthermore, a Finnish study suggested that fermented milk prepared with *Lactobacillus gasseri *TMC0356 could alter serum IgE concentration through a Th1 immune response in subjects with perennial allergic rhinitis[57]. Others reported that the ingestion of *B. longum *reduced ocular and nasal symptoms as well as need for medication, again in Japanese cedar pollinosis [55,56,58,59]. With regard to house dust mite allergy, the use of probiotics resulted in a reduction of symptoms in children and adults with allergic rhinitis [60,61]. In a recent study by Giovannini [52], *L. casei *DN-1 14 001 reduced the number of rhinitis episodes in sixty-four pre-school children with allergic rhinitis. Other studies did not show benefit, for example, patients who were allergic to birch pollen and apple food that were treated with *L. rhamnosus *GG during the birch-pollen season, had no reduction of symptom score, nor of sensitization to birch pollen and apple after *Lactobacillus rhamnosus *supplementation [62] and *L. casei *strain Shirota did not reduce allergic symptoms of Japanese cedar pollen allergy [63].

## Summary

Probiotics may have a potential role in the prevention and treatment of atopic dermatitis, but studies to date have not been conclusive. Parents should be aware that unequivocal benefit remains to be found. However, the effect can be modest and may depend on the target population. The data addressing the effect of probiotics in allergic rhinitis is even less robust.

Currently, there is no role for probiotic therapy in the treatment of asthma. Future studies will be important to refine the current knowledge base for potential use of probiotics in allergy.

## Competing interests

The author declares that they have no competing interests.
